# An Essay on Venereal Diseases, and the Uses and Abuses of Mercury in Their Treatment

**Published:** 1831

**Authors:** 


Art. XI. *An Essay on Venereal Diseases, and the Uses and Abuses of Mercury in their treatment. Illustrated by drawings of the different forms of Venereal Eruptions.* By Richard Carmichael, M.R.I.A, Vice-President of the Royal College of Surgeons in Ireland, and one of the Surgeons of the Richmond Surgical Hospital, Dublin, &c. &c. &c. *With Practical Notes, &c.* By G. Emerson, M. D. Philadelphia: Judah-Dobson and A. Sherman, 1825.

Hennen's *Military Surgery*—observations on Syphilis.

Most of our readers are aware of the investigations which have lately been made upon the subject of syphilitic and venereal diseases, and how completely these researches have eventuated in the overthrow of principles which, under the authority of Mr. Hunter, have governed the practice of physicians since his day.

The first of the writers at the head of this article, attempts a more full and definite classification of the diseases which resemble syphilis, and which have hitherto been confounded with it. Mr. Hunter was led from his own observations to suspect that many affections resembling syphilis, both in the primary and secondary symptoms, and in their general course were in reality very distinct diseases. The enquiry was extended by Mr. Abernethy, who added many new observations and threw great additional light upon the subject; but the principles at which these gentlemen arrived were necessarily defective and indefinite, on account of their assuming mercury as the test of syphilitic diseases.

Mr. Carmichael while surgeon to the Lock Hospital in Dublin, enjoying ample opportunities for an investigation of this kind, devoted himself assiduously to ascertaining the nature of the pseudo syphilitic diseases, and the relation in which they stand to syphilis, and the other contagious diseases.

The fact that the various exanthematous diseases, not only the small pox, and the measles, but the yaws a disease common among the slaves in the West Indies, the sivvens a disease frequently met with in Scotland, and other analogous diseases met with among the natives of Sweden, Canada, and Ireland, pursue a uniform course, would justify us in expecting, a priori, that the varieties of the venereal disease do not arise from a single poison whose effects are modified by accidental circumstances growing out of the constitution, climate, mode of life, &c. In accordance with this presumption Mr. Carmichael thinks he has established the fact, that there are three varieties of the venereal disease, arising from a specific infection, and running through a distinct and well defined train of symptoms, that are very different from those of the true syphilitic disease.

In selecting a symptom for the purpose of characterizing these forms of disease he has fixed upon the cutaneous eruptions, as less liable to be affected by constitutional derangement, local irritation, or errors of treatment.

The principal objection to this classification is, that the secondary symptoms upon which it is founded, in a majority of cases, do not occur. The primary ulcers, form a ground of distinction less accurate, but when unaltered by irritation or mercury, sufficiently certain to enable us to predict the nature of the eruption likely to follow them. The ulcers in the throat, from their uncertainty, he rejects from the diagnosis almost entirely,

The following synopsis gives his classification and his view of the treatment appropriate to each variety.

---

### SYNOPSIS.

### I

### PAPULAR VENEREAL DISEASE.

| *Primary Symptoms.* | *Remedies.* |
|---|---|
| 1. The simple primary ulcer,<br>2. Patchy excoriation, attended with a discharge, | Astringent washes.<br>Antimonials.<br>Purgatives. |
| 3. Gonorrhœa virulenta, | Astringent washes.<br>Antimonials. Terebinthinates. |
| 4. Buboes, | Leeches. Cold applications.<br>Blisters. Poultices. |

| *Secondary symptoms.* | |
|---|---|
| 1. Papular eruption, preceded by fever, and ending in desquamation,<br>2. Ærithematous inflammations of the fauces,<br>3. Swelling of the tonsils and glands of the neck,<br>4. Pains in the larger joints resembling rheumatism. | General blood letting to be adopted when indicated by the fever, and proportioned to its degree.<br>Antimonials. Sarsaparilla.<br>*Mercury unnecessary in any stage; and highly injurious until the eruption desquamates, the fever is subdued, and the disorder is evidently on the wane; and then an alterative course of antimony and calomel may occasionally accelerate the cure.* |

| | |
|---|---|
| 5. Iritis. | *Full mercurial affection of the system until the inflammation is subdued.* Local bleeding. Blistering. |

---

### II.

### PUSTULAR VENEREAL DISEASE.

| *Primary Symptoms.* | *Remedies.* |
|---|---|
| 1. The ulcer with elevated edges, without induration. | Astringent washes. Antimonials. Purgatives. |
| 2. Buboes. | The same treatment as for the buboes in No. I. Excision of the undermined edges of the Bubo. |
| *Secondary Symptoms.* | |
| 1. Eruption of pustules in general phlyzacious, preceded by fever, and terminating in ulcers covered with thin crusts that heal from their margins, and when the disease is on the wane, the eruption desquamates into scaly red blotches. | General blood-letting, as in No. I. Antimonials. Sarsaparilla. Guaiacum. Tar Ointment. Baths of sulphurated kali. Sulphureous fumigations. Nitro-muriatic acid baths. *Mercury is decididly pernicious until the pustules terminate in scaly blotches instead of forming ulcers; and then mercurials, in alterative doses, conjoined with sarsaparilla or guaiacum, may occasionally be employed with benefit.* |
| 2. Ulcers on different parts of the fauces, in general of a white apthous appearance. | The same general treatment, conjoined with the following local applications: Common detergent gargles. Mercurial gargles. Oxymel Æruginis. Fumigations of Hydrarg. Sulphuret. rub. or of Hydrarg. cum Creta. |

| | |
|---|---|
| 3. Pains in the joints. | The same general treatment, conjoined with the following local applications:<br>Leeches. Fomentations.<br>Bread and water poultices.<br>Blisters. Ointment of tartarized antimony.<br>*Mercury is to be particularly avoided while inflammation oj the knee exists.* |
| 4. Nodes. | The same general treatment, conjoined with the following local applications:<br>Leeches. Bread and water poultices.<br>Blisters. Ointment of tartarized antimony.<br>Division of the periosteum.<br>*If the preceding remedies prove inefficient, mercury may be of advantage, provided the general disease is on the wane.* |

---

### III

### PHAGEDENIC VENEREAL DISEASE.

| *Primary Symptoms.* | *Remedies.* |
|---|---|
| 1. The phagedenic ulcer,<br>2. The sloughing ulcer. | Bread and water poultices with opium occasionally added.<br>Warm fomentations.<br>General blood-letting, proportioned to the acuteness of pain, inflammation, and symptomatic fever.<br>Antimonials in nauseating doses.<br>Opium, Cicuta, Hyoscyamus } in full doses.<br>Purgatives. |
| 3. Buboes. | The same treatment as for buboes, in No. II. |

| *Secondary Symptoms.* | |
|---|---|
| 1. Eruption of tubercles, or spots of a postular tendency, or both intermixed, preceded by fever, and terminating in ulcers covered with thick crusts, which often assume a conical form, healing from their centre, and extending with a phagedenic margin. When the disease is on the wane, they desquamate like those of No. II. into scaly red blotches. The disease is attended with great debility, particularly when the ulcers are extensive. | General blood-letting, as in No. I and II.<br>Antimonials.<br>Sarsaparilla.<br>Guaiacum.<br>Compound powder of ipecacuanha.<br>Opium.<br>Cicuta.<br>Nitrous acid.<br>Nitro-muriatic acid bath.<br>*Mercury, (with the exceptions noticed below) increase the ravages of the disease in all its stages, but the last. It may theu be given with safety and advantage, as in No. II.* |
| 2. Ulcers on different parts of the fauces, and particularly the back of the pharynx, frequently engaging the entire fauces, and sometimes extending to the larynx and nares. | The same general treatment, conjoined with the following local applications.<br>Oxymel Æruginis.<br>Strong solution of muriate of mercury, or nitrate of silver.<br>Fumigations of Hydrarg. Sulphuret. rub. or of Hydrarg. cum Creta.<br>*If these should prove inefficient, mercury may be used largely with advantage in checking the progress of the ulceration, even though it should exasperate the general disease.* |
| 3. Ulceration and thickening of the larynx. | Caustic issues or applications of moxa on each side of the thyroid cartilage. Tracheotomy. |
| 4. Ulceration, and caries of the nose. | The same general treatment, with the yellow and black mercurial washes frequently injected into the nostrils.<br>Fumigations of the Hydrarg. Sulphuret, rub. or of the Hydrarg. cum Creta through the nares.<br>*If these remedies fail, mercury may be used as above.* |
| 3. Pains of the joints. | The same treatment as in No. II. for the same symptom. |
| 6. Nodes. | The same treatment as in No. II. |

### IV

### SCALY VENEREAL DISEASE.

| *Primary Symptoms.* | *Remedies.* |
| --- | --- |
| 1. The chancre, or callous ulcer.<br>2. Buboes. | *Full courses of mercury for both primary and constitutional Symptoms, except a tendency to phthsis pulmonalis or other delicacy of constitution should forbid them* |
| *Secondary Symptoms.* | |
| 1 Eruption of Scaly blotches; presenting either the character of lepra, or psoriasis, and unattended with any obvious degree of fever. | *Mercury.* |
| 2. Excavated ulcers of the tonsils.<br>3. Pains in the joints. tibiæ, cranium, &c.<br>4. Nodes. | The same local remedies as prescribed for the several symptoms in No. II. |

The ulcer which exists as the primary symptom of the papular form of the disease, is a simple ulcer, distinguished by negative characters—without induration, elevated edges, or phagedena. These ulcers gradually rise above the surrounding skin, exhibit a smooth surface of a healthy color, but without granulations, and of a fungous appearance. They cease to spread as soon as the fungous stage commences, and the average period of their continuance is from three to six weeks. They are more frequently met with upon the glans, or the internal surface of the prepuel, in which situation they frequently occasion phymosis, but they often appear also on the body of the penis, or the scrotum, in which last situation they are so much elevated as to resemble fungi or soft warts.

From the simultaneous occurrence of two or even all three of these primary symptoms, Mr. C. infers that they arise from the same poison. This opinion is confirmed by the fact, that the same papular eruption follows them, and that men have caught the two first symptoms from those who exhibited no other form of the venereal disease than the last.

Mr. C. attempts to account for the fact of gonorrhœa not occasioning secondary symptoms more frequently by the analogy of the variolous and vaccine disease, in which we observe that, as the fluid in the pustule assumes the form of pus, it loses its specific character, and either fails to communicate the disease, or imparts to it one of a specific character. He therefore considers the facility with which the mucous surfaces take on inflammation, as a provision of nature to supply the defect of a cuticle, and prevent the introduction of poisons into the system. Mr. Travers, who admits that secondary symptoms arise from gonorrhœa, thinks they never take place so long as the surfaces preserve their integrity, but occur only as a consequence of ulceration of the mucous surface.

The following extracts will give an idea of the constitutional symptoms:

"The CONSTITUTIONAL SYMPTOMS of the papular venereal disease consist of more or less of fever, attended with pain in the head, shoulders, and larger joints; and sometimes pain in the chest, with considerable dyspnœa, which ushers in an eruption that chiefly appears on the forehead, chest, and back, but also extends in a more scattered way over the extremities.

The fever does not subside on the appearance of the eruption, although it is at its height just previous to that event. It exists as long as fresh crops of the eruption continue successively to appear, and is usually accompanied with pains of the several joints, which are most severe at night.

The papulæ vary from a pale red to a deep crimson. Some of them are simply pimples, while others are almost advanced to the pustular form. * * * They do not all make their appearance together, as in the eruptions of the exanthemata, but follow each other in succession; so that on the same patient some spots will appear in their commmencement like small pimples; others, which have arrived at maturity, form larger pimples, with acuminated tops, containing pus or lymph; while others, on their decline, consist of exfoliations of the cuticle. Their color, in their latter stages, becomes paler, and assumes a copper tint, while the exfoliation of the cuticle gives an appearance of scaliness, a state in which it is most liable to be confounded with the scaly eruption of syphilis. But they may be readily distinguished from each other; for when the papular eruption is on the decline, and has assumed a pale red or copper color, on examining the patient, we shall find other spots in their papular or pustular form.

which will at once point out the character of the eruption. But the very appearance of the declining papulæ will, to a discriminating eye, be sufficient for this purpose; for its copper-colored scaly surface is more raised in the centre than its circumference, while the reverse is the case in the scaly leprous eruption of syphilis. * * * The soreness of the throat is different from that which takes place in syphilis, in which there is a deep excavated ulceration of the tonsils, with little inflammation or difficulty of deglutition. In this disease, on the contrary, the patient complains of considerable soreness, and difficulty of swallowing; and, on examination, the entire fauces, but more particularly the back of the pharynx, exhibit an erithematous appearance, and not unfrequently with considerable swelling of the tonsils, which assume, as they always do when swelled, an irregular appearance, that is often mistaken for ulceration. The cervical glands also frequently swell and ulcerate in this disease, but more frequently when the eruption is on the decline. * * * The eruption, after having wholly disappeared, will, in some few instances, return again and again, at uncertain intervals, of from one to several weeks, each successive crop, however, being less than the former, and attended with less constitutional derangement. The intervals between those attacks are also greater, as the disease exhausts itself, or yields to the powers of the constitution. But if the progress of the disease has been interrupted by mercury, before it has arrived at its latter stages, it becomes more obstinate and complicated than it would otherwise have been."

In the advanced stages of this form of the disease, especially if the eruptive fever have been superceded by mercury, or if the eruption have been repelled by cold, inflammation of the iris is a very common attendant. The author states that, in the whole course of his practice, during several years of which he has been surgeon to a hospital appropriated exclusively to venereal cases, containing an average number of from 280 to 300 patients, and also to an extensive eye dispensary, he has seen but one case of venereal iritis accompanied by an eruption, in which that eruption was not papular.

One of the characteristics of this form of venereal disease is the absence of nodes. In a few cases, swellings do indeed occur in the course of the tibia, but they differ from nodes in possessing more of the inflammatory character, and affecting the integuments and not the bone, appearing suddenly, and disappearing as rapidly, without the exhibition of mercury.

With regard to the treatment of this papular form of the disease, our limits permit us to do little but refer our readers to the synoptical view. In this, which Mr. C. considers by far the most frequently met with, embracing, in his opinion, at least three-fourths of the patients, mercury is useful merely as an adjuvant in the advanced stages, and then, only as an alterative. The following extract wlll give a more extended view of the author's general treatment.

"In fine, our object in the treatment of the constitutional disease under consideration should be,—1st. To moderate the action of the system, if the fever which attends and accompanies the eruption should be violent.—2dly. After the fever is considerably lessened, or subdued, the exhibition of sarsaparilla, either alone or combined with antimonials, affords the most safe and efficacious mode of treatment. The action of sarsaparilla, particularly when assisted by antimonials, is to increase all the secretions; and these must not be checked, particularly that of the skin, by imprudent exposure to cold. The diet of the patient may be light and nourishing, but not heating or stimulating, and he ought to increase the quantity of mild diluting drinks he is in the habit of taking, which will assist the action of sarsaparilla on the skin and kidneys. The practitioner himself should be observant that this medicine is carefully prepared, and of a good quality. Not only boiling water extracts the virtues of sarsaparilla, but lime-water is supposed, by acting on the cortical part, to be equally efficacious.

3dly. When the eruption has declined, no new spots appearing, and and those that remain all desquamating or scaly, while the patient still complains of lingering pains in his head, elbows, hips, and knees, the disease being obviously on the wane, it may now be subdued altogether by alterative doses of mercury conjoined with antimony, for which object the compounded calomel pill of the London Pharmacopœia affords an excellent example; and with this medicine the sarsaparilla may still be continued, either in the form of decoction or infusion."

We make one more extract to impress upon our readers the diagnosis between the papular venereal, and the syphititic diseases.

"In the papular venereal disease, the eruptive fever is more strongly marked than that of syphilis; and, in many cases, is so acute as to require repeated blood-lettings. The eruption is not scaly, like that of syphilis, but papular, disappearing and recurring repeatedly. The affection of the throat is not a deep ulceration of the tonsils, but an inflammation and superficial excoriation, or rawness of the fauces, but particularly of the posterior part of the pharynx. The pains do not affect the centre of the long bones, but the large joints. This disease is not attended with nodes, but inflammatory swellings of the integuments have been mistaken for them, which occur suddenly, and as suddenly disappear, without the assistance of mercury."

The 4th chapter of Mr. C's work is devoted to *the pustular venereal disease.* This disease, Mr. C. remarks, both in its primary and secondary symptoms, forms a link between the papular and phagedenic venereal diseases, being more obstinate than the one, and destitute of the malignity and destructive tendency of the other. The following is his description of the primary symptoms.

"The primary ulcer of this disease is characterized by a reddish brown surface, which borders closely on the phagedenic character. Its edges are raised, and well defined. It is not excavated, but is either on a level with the surrounding skin, or considerably raised above it. At its commencement it appears in the form of a small pustule, attended with itchiness of the part.

It may be distinguished from the primary ulcer of the papular disease, by its well defined and elevated edges, and also by the absence of the smooth fungous appearance which characterises the second stage of that ulcer. From the phagedenic primary ulcer to be described in the next chapter, it may be distinguished by its well-defined margin, and from the absence of the irregular and corroded-like surface of that ulcer, and also by its exemption from the painful and acute symptoms by which the phagedenic ulcer is characterized. It may also be distinguished from chancre by its want of the callous edge and base which always attend that ulcer. The ulcers of which we are treating, are most frequently found on the external surface of the prepuce, body of the penis, and occasionally on the scrotum, and occur from the size of the smallest pea, to that of a shilling. Of the former size they frequently form a circle round the orifice of the prepuce, and occasion, when the ulcers heal, a permanent phymosis, which can only be removed by the knife. They are, in general of a chronic nature, and evince but little disposition to spread."

The buboes arising from this virus resemble the primary ulcers in their tendency to form projecting or undermined edges, which are so very indolent that the most powerful caustics are insufficient for their removal, and Mr. C. invariably resorts to the knife for that purpose.

Full courses of mercury always increase their disposition to *burrow.* Stimulating applications to the ulcers do no good, and if the ulcer is irritable, encourage it to extend. Rest, with gentle astringent applications, or mild ointments are all that is requisite.

This form of venereal disease frequently manifests a disposition to run into the preceding one; the pustules are frequently combined with papulæ, which terminate in desquamation instead of the superficial sores covered with thin scaly crusts, which characterize the pustular form of the disease. And Mr. C. remarks, that the more closely the eruption resembles the papular variety, the more mild, manageable, and yielding will the disorder be found.

Chapter V. *Phagedenic Venereal Disease.* The primary symptoms of this form of the disease are the *phagedenic ulcer*, and *the sloughing ulcer*, of which the author gives the following description.

"The phagedenic ulcer, as its name implies, has a corroded appearance, and neither exhibits granulations, or surrounding induration. It spreads sometimes with rapidity, causing the most destructive havoc in the course of a few days; and in other instances creeps on slowly, healing in one part and making progress in another; but unlike a chancre, instead of being checked by mercury, it is almost always rendered more inveterate and rapid in its progress by that mineral. It more frequently attacks the glans penis than any other part; but the ulcer usually proceeds to affect the prepuce, which it often entirely consumes, and continuing its depredations on the corona and glans, at last effects their total destruction. When this event takes place, the ulceration usually receives a sudden and permanent check. At other times, a spontaneous hemorrhage, owing to the destruction of the coats of an artery, occasions a favorable change. More rarely it happens, that notwithstanding every anodyne and lenient application, the ulceration will gradually proceed, until the entire penis is destroyed. There is also another characteristic of this ulcer worthy of remark, viz., the frequent return of ulceration, after the part has healed, to the very same spot which was at first affected.

The sloughing ulcer is still more untractable and destructive than the phagedenic: but the surgeon has seldom an opportunity of seeing its commencement, as the disease excites so little uneasiness at first, that, in general, its attack is unobserved even by the patient, until it has existed for several days. A small black spot that resembles a grain of shot in color as well as in size, is its first appearance; which if seen by the experienced eye of a surgeon, even at this early period, will at once be recognized as a slough or mortification, extending to some depth below the surface. The slough will continue to increase, sometimes, to only three or four times its original extent, and at others until it engages a considerable portion of the penis, before a line of separation can be observed between the living and the mortified parts. When the separation at length takes place, we do not find a clean granulating sore, as occurs in simple mortification; but a corroding phagedenic ulcer, which begins a new kind of depredation on the surrounding parts, equalling the virulence but not the rapidity of the sloughing process by which it was preceded.

Suddenly those parts are attacked by severe pain, and afterwards assume a bluish cast, and on the following day they are found to be covered by a slough; and in this way this destructive malady continues to extend its ravages by alternate sloughing and ulceration, until in one sex the entire penis, scrotum, perineum and pubes are destroyed; and in the other, until the labia, nymphæ, vagina, anus, nates, and I believe, even the bladder and uterus, are engaged in one extended and malignant state of putrefaction."

The author remarks that it is often difficult to determine with precision the character of these ulcers, since the improper exhibition of mercury, neglect, or irritation, may cause any venereal disease to slough, and ulcerate. It is therefore of the utmost importance to ascertain whether this disposition existed from the commencement, or has arisen from some accidental circumstance.

Buboes rarely attend this form of venereal disease. Before ulceration, they exhibit no peculiar characters, but afterward they partake of the general malignity of the disease.

"The edges of the ulcer become undermined, jagged, and irregular; and there is no chance of inducing them to cicatrize until these projecting edges are removed, which may be done either with caustic or with the knife; the latter is the mode I always prefer as the least painful and tedious."

The constitutional symptoms are

"*First*, An eruption of tubercles, pustules, or spots of a pustular tendency, which quickly degenerate into ulcers covered with thick crusts, that usually heal in a peculiar manner from the centre, while at the same time they are extending at their circumference with a phagedenic border. This eruption is often ushered in by a high degree of fever, which is lessened, but not altogether removed on the appearance of the eruption. Sometimes the patient will complain of being generally unwell, for a considerable period preceding the eruption, without being able to state any particular symptoms to account for his indisposition: however, a general listlessness, pallid counte nance, languid eye, and broken rest, sufficiently evince that he is neither fanciful or hypochondriacal.

In other instances, severe nocturnal head-achs, with tenderness of the scalp, or slight dyspnœa, with tenderness of the sternum upon pressure, and general soreness of the chest, afford to the wary practitioner the first intimation that a venereal taint may be present, and induce him to make further enquiries bearing upon this point. The premonitory symptoms may undoubtedly usher in the eruption of any of the other forms of venereal disease, but repeated observation enables me to state, that they are more severe in the phagedenic, than in any other form of venereal complaint."

Mr. C. thinks it probable that the reason that these ulcers heal from the centre is, that the skin being destroyed, the cellular membrane not being involved in the disease, throws out granulations, which cicatrize as under other circumstances.

"*Second.*—The ulceration of the throat which attends the phagedenic disease, is of a most formidable nature; according to my experience, it commences in the form of a small white apthous-looking sore, which usually attacks the velum, or posterior part of the pharynx, but more frequently the latter. It sometimes, but more rarely, commences in other parts of the throat. If not checked in its progress, wherever it may arise, it rapidly engages the entire of the pharynx; extending upwards, the nares become affected, followed too frequently by caries, and exfoliation of the spongy bones, and tenderness of the ossa nasi, with a foul discharge from the nostrils. This ulcer, in its progress towards the mouth, also affects the tonsils with a similar ulceration; and seizing upon the velum, and uvula, rapidly destroys them. So that in looking into the mouth of a person in this lamentable state, there appears one vast continuous ulcerated cavity, covered with a white viscid matter, and extending from the palate to the lower part of the pharynx."

Mr. C. has never witnessed a recovery where the larynx was decidedly attacked with ulceration; the patient invariably dying, with all the concomitants of phthisis. The disease frequently extends to the nose, causing exfoliation of the bones, and sinking in of the cartilages. All the cases in which Mr. C. has witnessed the affection of the nose, have been of this or the preceding variety, principally of this. He has never seen it occur in the papular or true syphilitic variety, and he attributes the frequency of this affection in Portugal, to the prevalence of the phagadenic disease, in that country.

"*Third.*—At the same time that the patient is affected with the eruption and ulceration I have described, he is in general attacked with severe and obstinate pains in his joints; particularly in his knees, wrists, and ankles, which often become swelled, red, and so painful, that they are highly sensible to the slightest touch. The affection of the knee is usually attended with considerable swelling, and every symptom that denotes an acute attack of inflammation of the synovial membrane. This affection of the knee is so frequent an occurrence, in this form of venereal disease, that it may be almost esteemed one of its peculiar characters."

*Fourth.* Nodes in this disease have only been traced to such cases as have been treated with mercury. A variety of other anomolous symptoms have also been noticed, but the author professes himself unable to decide whether they are justly attributable to the disease, or whether they are to be considered as complications produced by the use of mercury.

Mr. C. has no doubt that the embarrassing obstinacy of this disease arises from the indiscreet use of mercury. In other eruptive diseases we remark that the cutaneous affection relieves the oppression of the vital organs; and he therefore infers, that when the syphilitic eruption is prematurely cured by mercury, the disease is transferred to the deep seated parts; the periosteum, the fascia, and the bones, and that this is the true reason why the patient so often suffers from diseases of these parts, after he has undergone a full course of mercury, and flattered himself from the disappearance of the disease of the skin, that the disease was eradicated from the system..

The phagedenic ulcer, in its active state, will generally yield to means calculated to remove pain and inflammation, absolute rest, venesection, nauseating doses of antimonials, warm poultices and fomentations, opium, circuta, and hyoscyamus, until the inflammatory symptoms are subdued. Afterwards, a weak lotion of nitrate of silver, the black or yellow mercurial washes are sometimes useful.

Stimulating applications, such as Venice turpentine or balsam copaivæ combined with olive oil, or the tincture of myrrh combined with six or seven parts of the camphorated mixture, are of advantage by stimulating the sound parts to cast off the sloughs, but they do not prevent them from being renewed.

Bark is always injurious, causing the ulcer rapidly to extend. Indeed, by the high degree of fever present, and from the relief produced by spontaneous hemorrhage, a contrary practice appears to be strongly indicated. Opium and cicuta in large doses are frequently decidedly useful.

The secondary symptoms are to be treated on the same general principles as the primary; by blood-letting, laxatives, antimonials, blisters, if the pain in the head be severe, rest, low diet, diluting drinks, and sarsaparilla. Guiacum he considers useful, but inferior to sarsaparilla. For obvious reasons, these remedies should not be used in very cold weather, unless the patient will confine himself to the house. When the cutaneous affection was tubercular, and disposed to spread into foul ulcerations, he found the nitrous acid very useful in the proportion of two drachms of the diluted acid to a quart of the decoction of sarsaparilla, of which the patient is to take as much as will agree with his stomach. These remedies are to be variously combined with each other, and with opium and cicuta, according to circumstances.

It is particularly to be recollected, that the author considers mercury, in every form and every proportion, decidedly injurious, until the inflammatory symptoms begin to subside, and the constitutional ulcers to heal.

When the ulcers in the throat are spreading with alarming rapidity, Mr. C. resorts to fumigations with the sulphuret of mercury. This, he remarks, is apparently inconsistent with his general principles; but as the least of two evils, he thinks it better to arrest the spread of the ulcer, though at the risk of exasperating the general disease. So great is the advantage derived from this application, that the ulcers are always arrested, if the disease have not extended to the larynx.

When the nares become affected, the author recommends the local application of mercury, by a mode original with himself; either by anointing the affected nostril with citrine ointment softened with olive oil, or a weak mercurial wash, made by adding half a grain of oxymuriate of mercury, to an ounce of lime water. When the larynx is decidedly involved in the ulceration, the disease has hitherto, in the author's practice, terminated fatally with the symptoms of consumption.

When the joints become affected, the antiphlogistic regimen, local depletion, poultices, and warm fomentations, are to be resorted to. When the affection becomes chronic, there is no application equal to the tartar emetic ointment, used in conjunction with the warm bath and the constitutional treatment already detailed.

For nodes, he recommends leeches followed by blisters, and if these fail, mercury, notwithstanding they may have been occasioned by the abuse of this very remedy.

Chapter VI. is devoted to the true syphilitic disease. The true Hunterian chancre, the description of which is so well known to every student of medicine, the author states, is invariably followed by the scaly venereal disease, the description of which is repeated by every writer on this disease, and with which we shall not therefore trouble our readers. This form of the disease, Mr. C. alleges has become much more rare of late; so much so, that since the publication of Messrs. Rose & Guthrie, who advocate the non-mercurial practice, the author, although surgeon to a large hospital, and engaged in extensive private practice, has not met with more than six cases of true chancre, upon which to test the new mode of treatment. There are few ulcers, he remarks, of an irritable nature, which have not some degree of fulness around them; but this fulness is very different from that callosity which Hunter describes. The result of his trial in these few cases is this: that although this form of the venereal does not absolutely require mercury for its cure, yet it differs from them all, in being decidedly benefitted by mercury in all its symptoms and stages.

With regard to the accuracy of Mr. Carmichael's classification, and the truth of the opinion that the different forms of the disease arise from a distinct virus, it will not be expected that the opinions of the profession can be very unanimous, at a time when so many are disposed to doubt the existence of a specific virus altogether. There is a very great difference of opinion upon this subject; some referring the difference of symptoms to constitutional or epidemic causes, or to both; others admitting the conclusions of Mr. C. to a greater or less extent.

Whatever may be the fact respecting the specific virus, a considerable point would be gained if it could be proved that the same causes which produce one variety of primary ulcer, invariably produce the form of secondary disease, which Mr. C. has connected with it.

Upon this subject the following quotations from Dr. Hennen would lead to a very diffierent conclusion.

"The appearances of the primary sores contracted by sexual intercourse, which have presented themselves in the military hospitals, have varied extremely, but in many instances they have been very much influenced by their particular position. The following circumstances have been principally remarked in them: 1st, Ulcers on the external integuments have generally had round callous edges, level surfaces, but little induration of base; they were less irritable than others, became sooner clean, and healed uniformly, but slowly. 2d, Ulcers on the internal membrane of the prepuce, have been generally either superficial or elevated; their surfaces covered with a light colored slough, or of a bright red, with villous appearance; their edges either regularly defined, or spread out like excoriations; their bases have been, in general, but little indurated, but when the ulcers have spread out, they have sometimes acquired a cartilaginous hardness, and have been extremely difficult to heal. 3d, Ulcers immediately behind the corona glandis, have been, in general, highly irritable, deep scooped, indurated in their edges and base, foul, with membranous bridles running across them, throwing off a perceptible slough; but if mildly treated, soon healing after that event. 4th, Ulcers on the frœnum have generally followed lacerations of that part, have had considerable induration of base, and have been generally slow of healing. 5th, Ulcers of the glans have been generally excavated, but with little hardness of base; quickly throwing off a slough, and then healing rapidly.

It has sometimes happened that where a sore has spread and occupied different textures, each of its parts has exhibited the character which has generally prevailed in sores confined to that particular texture. Thus, in a sore which has implicated part of the internal prepuce, corona, and glans; on the first spot it has been elevated, on the corona it has been indurated and irritable, and on the glans excavated, but with little hardness. * * * *

We have had frequent opportunities of remarking two or more sores of different kinds existing at the same time; an irregular shaped diffused sore; an elevated sore, covered with a light colored slough, as if a bit of shamoy leather had been stuck on by some tenacious substance; a groove or streak along the glans, as if made by a scraping instrument, filled with purulent matter; and the true and perfect chancre, according to Mr. Hunter's definition, or the true syphilitic ulcer, according to Mr. Carmichael. This last has in some cases occupied the glans, in some the prepuce, while the sores of another description have been on the same part close beside it, or on another part at a distance. Three of these cases I particularly selected for examination and public demonstration in the castle hospital; in one, the Hunterian chancre was on the glans, and a sore without any hardness on the prepuce; in another, it was on the prepuce and a simple ulcer on the glans; in the third, a most perfect specimen of Hunterian chancre occupied the internal prepuce close to the corona glandis; and at about half an inch from it, nearer the frœnum, but farther from the glans, was an elevated ulcer: in all these cases the Hunterian chancre healed several days before the others.

In fifteen cases of eruptions unaccompanied by any other symptoms, which succeeded the Hunterian sore, six were tubercular, five exanthematous, two pustular, one tubercular and scaly, and one tubercular and vesicular.

In four cases following the same sore, but in which the eruptions were complicated with sore throat, two were tubercular, one was tubercular and scaly, and one was tubercular and exanthematous.

In twelve cases following the *Non*-Hunterian sore, and in which eruptions were the only symptoms, six were pustular, three were exanthematous, two were tubercular, and one was tubercular and scaly.

In seven cases where the eruption was accompanied with sore throat, three were exanthematous, two were tubercular, one was papular, scaly and tubercular, and one was pustular and tubercular. An examination of the table will show the existence of other morbid combinations.

The eruptions, after having disappeared, have, in some cases, again occurred a second and third time, and in a different form from the original attack; cold and excess were the causes to which this re-occurrence has been principally traced. In these relapses, the mottled, the papular, and the pustular, have been the most common forms, and have in general preceded the scaly and the tubercular eruptions. The scaly, although in some instances it has been from the first under the form of psoriasis, or branny scurf, has been in general a degeneration of the papular, and occasionally it has appeared on the apices of the tubercular, or was intermixed with them."

We shall devote the remainder of this article to the non-mercurial mode of treating syphilis. Practitioners have existed at all times, who have asserted the possibility of curing this disease without mercury, and who have not failed to attribute all the unpleasant consequences which result from this disease, to the abuse of that remedy. The result of the experiments which have been made of late years, have confirmed these views, and proved, that in none of its forms is mercury indispensably requisite—a fact certainly, very little to the honor of a profession which has hitherto almost unanimously considered the curative influence of mercury a test of syphilis.

But although the possibility of curing the venereal disease without mercury should be established, it would by no means follow that it was expedient to dispense with it under all circumstances. The determination of this question would involve a variety of considerations, of which the following may be considered the most important. 1. The comparative length of time required for the cure of the primary symptoms under the two modes of treatment; 2. The comparative frequency of the occurrence of secondary symptoms; 3. The comparative severity of the secondary symptoms; 4. The comparative length of time required for the cure of the secondary symptoms; 5. The effect upon the constitution of the different modes of treatment.

Upon this last head we will remark, that while mercury is the most powerful remedy yet discovered against numerous inflammations, yet if given incautiously, and in some constitutions if given with every possible precaution, it tends to aggravate, and sometimes to establish constitutional diseases, to undermine the healthy constitution of the body, to increase constitutional irritability, to excite inflammation and ulceration in the mucous textures, to produce marasmus, and to destroy the texture and healthful proportion of the osseous system, and to induce dropsical infusions in the synovial and serous cavities,—in a word, to produce effects far more dreadful than the worst primary or secondary effects of syphilis.

Upon the other points we will refer to a report of the Army Medical Department of Great Britain, of which the following is a summary.

*Army Medical Department, April 2, 1819.*

*Circular.*

ON SYPHILIS.

In transmitting the following summary of the conclusions on the question of syphilis and its treatment, we have to assure all, that it may be considered as an unprejudiced statement drawn up from the answers *alone* of the regimental surgeons, to the queries transmitted by us to them in December last.

*Without Mercury.*

1st, That since December, 1816, to December, 1818, there appears to have been treated for primary venereal ulcerations on the penis, (including not only the more simple sores, but also a regular proportion of those with the most marked characters of syphilitic chancre, as described by Hunter and other writers,) 1940 cases.

2d, That of these 1940 cases so treated, 96 have had secondary symptoms of different sorts.

3d, That in these 96 cases of secondary symptoms following sores treated without mercury, it was deemed necessary to have recourse to mercury for a cure for twelve of them, for which change the following different reasons are assigned in different cases by the surgeons who treated them.

*a.* On account of sloughing ulcers in the throat.

*b.* The protraction of cure beyond the third week.

*c.* Because the general health seemed to suffer.

*d.* With a view of expediting the cure.

*e.* The reappearance of eruptions, or aggravation of symptoms.

*Note.*—In several of these 12 cases, alterative doses of mercury were sufficient to effect the cure.

4th, That in 1940 cases of primary symptoms treated without mercury, (as described in part 1st,) its use was resorted to in 65 of them; the reason assigned being as follows:—

*a.* An indisposition to yield to the local application in three weeks.

*b.* The sore spreading.

*c.* The appearance of fresh sores.

*d.* Buboes suppurating, and not disposed to heal.

*e.* The general health appearing to suffer.

*f.* A belief that the constitution became affected from the continuance of the sores.

5th. That these 1940 cases, treated as here above stated, are now recovered of their venereal complaints," and either doing their duty as soldiers, or have been discharged for military reasons totally unconnected with venereal disease.

6th. That the principal remedies employed have been (speaking in general terms, and with reference to primary sores) confinement to bed in many cases, in all to hospital, spoon diet, occasionally general bleeding when inflammation ran high, (in six or eight cases,) purgatives, antimonials, pretty generally emollient soothing applications in the first instance, generally cold or warm water, (the latter frequently injected between the prepuce and glans,) and the first externally applied, the water frequently mixed with the liquor plumbi; in the latter stages, the lotio hyd. submuriat., or muriat. in aqua calcis, lotio sulphat. cupri. argent., nitrat., &c., were employed. With reference to secondary symptoms, when mercury was not had recourse to, purgatives, antimonials, nitric acid, sarsaparilla, guaiacum in substance, or in combination with sarsaparilla, warm bath, nitro-muriatic acid bath, gargles, when the throat is affected. In nodes, fomentations, scarifications, leeches, and blisters.

7th, That the average period required for the cure of primary symptoms without mercury, when bubo did not exist, has been 21 days; with bubo, 45 days.

8th, That the average period for the cure of secondary symptoms without mercury, has been from 28 to 45 days.

9th, That every man treated without mercury has been fit for immediate military duty on dismissal from hospital.

*With Mercury.*

1st, That during the period specified before, there appears to have been treated of venereal ulcerations of the penis, (the characters given of which do not appear to have been, in any essential degree, different from those treated without mercury,) 2827.

*Note.*—" It may be perhaps well to view these as more generally bearing the character of Hunter's chancre."

2d, That of the 2827 thus treated and healed, 51 have had secondary symptoms.

3d, That there are good grounds for believing that, in the majority of instances, when secondary symptoms have occurred where the primary symptoms have been treated with mercury, that the secondary symptoms are more severe, and more intractible, than when mercury had not been used for the primary sores.

4th. That one man treated by mercury for primary sores, has been discharged the service on account of the injury his constitution sustained therefrom.

5th, That another man, after treatment for secondary symptoms by mercury, has been discharged the service in consequence of that complaint.

6th, That the average period occupied for the cure of primary symptoms without bubo, with mercury, has been 33 days, with bubo, 50 days, and that the great majority were fit for immediate military duty on dismissal from hospital.

7th, That the average period occupied in the cure of secondary symptoms, has been 45 days.

*Note.*—" The treatment by mercury is so generally known that it is deemed useless to describe it in either case." Much the same local applications were used in the treatment with mercury to the sores, as was described in that without it; perhaps more stimulating and escharotic applications were used, and less attention paid to regimen and diet, when mercury was given, at least less stress seems to have been laid on these.

*General Observations.*

1st, From the statement above made, it would appear that *all kinds of sores*, or primary symptoms of syphilis, may be cured (as far as a period of nearly two years will warrant the conclusion) without mercury.

It is considered that the exceptions noted in paragraph 4th do not present valid objections to the above conclusion, on viewing the general testimonies on this point; but to the reasons there assigned for the necessity of having recourse to mercury, the most particular attention is required, as on these must the propriety or impropriety of that measure depend.

2d, To guard against any fallacy in the comparative estimate of time employed in the cure of primary symptoms with and without bubo, it must be noticed that this is only an average statement; in some individual regimens, the period required without mercury has been longer than that with mercury.

3d, That it appears that the frequency or rarity of secondary symptoms would seem to depend on circumstances not yet sufficiently understood or explained, although the following fact would tend to the belief, that either the constitutions of the men, or the mode of conducting the treatment without mercury, are the causes that possess the greatest influence in their production.

In one regiment, four secondary cases out of twenty-four treated without mercury, supervened. In another regiment 68 cases have been treated within the specified time without mercury, all bearing marks of true venereal disease, (and 28 of these especially selected for their decided characters of chancre;) no secondary symptoms of any kind have hitherto made their appearance, and in all, fifteen months have elapsed since they were treated.

To this circumstance most particular attention is required, both with the view of ascertaining if peculiarity of constitution influences the appearance of secondary symptoms, and of pointing out the necessity of attending to the proper selection of local remedies adapted to the different stages and states of the sore, and to the general treatment of the constitution during the time the patients are in hospital, and that whether mercury be used or not.

4th, That it appears that no peculiar secondary symptoms are seen to follow from peculiar primary sores.

5th, It has been remarked, that in cases healed without mercury, iritis has been frequently observed as a secondary symptom, in some instances by itself, in others attended with eruptions of different kinds. In these instances, mercury has been generally resorted to with success.

6th, The reappearance of the primary ulcer, and repeated attacks of eruption, are the diseases which have been most frequently observed to succeed the non-mercurial practice.

7th, The conclusions arrived at by the additional testimony of many more regiments, not included in the number from whence this report has been drawn up, confirm, in every material circumstance, the results stated under both methods of treatment.

Accounts of other experiments, conducted on a very extended scale, confirm, in general, the above results, shewing that the average period of cause, both of the primary and secondary symptoms, is shorter, without mercury, and that the secondary symptoms are milder, though they generally occur more frequently.

It is doubtful whether the non-mercurial practice would be as successful in private as in hospital practice, since it is impossible to compel private patients to submit to the necessary restrictions. Enough, however, has been effected to shew, that in all cases in which any doubt exists as to the nature of the disease, or as to the propriety of resorting to mercury, the disease may safely be trusted to the natural efforts of the constitution, until the effect of diet, and mild treatment have been tried, and if an eventual resort to mercury should become necessary, that this remedy may be successfully used in such a manner as not to injure the most delicate constitution. F.

